# Symptom Experience, Management, and Outcomes According to Race and Social Determinants Including Genomics, Epigenomics, and Metabolomics (SEMOARS + GEM): an Explanatory Model for Breast Cancer Treatment Disparity

**DOI:** 10.1007/s13187-019-01571-w

**Published:** 2019-08-08

**Authors:** Maura K. McCall, Mary Connolly, Bethany Nugent, Yvette P. Conley, Catherine M. Bender, Margaret Q. Rosenzweig

**Affiliations:** University of Pittsburgh School of Nursing, 3500 Victoria Street, Pittsburgh, PA 15261 USA

**Keywords:** Breast cancer, Symptom, Social determinants, Treatment disparity, Chemotherapy, African-American, Dose intensity

## Abstract

Even after controlling for stage, comorbidity, age, and insurance status, black women with breast cancer (BC) in the USA have the lowest 5-year survival as compared with all other races for stage-matched disease. One potential cause of this survival difference is the disparity in cancer treatment, evident in many population clinical trials. Specifically, during BC chemotherapy, black women receive less relative dose intensity with more dose reductions and early chemotherapy cessation compared with white women. Symptom incidence, cancer-related distress, and ineffective communication, including the disparity in patient-centeredness of care surrounding patient symptom reporting and clinician assessment, are important factors contributing to racial disparity in dose reduction and early therapy termination. We present an evidence-based overview and an explanatory model for racial disparity in the symptom experience during BC chemotherapy that may lead to a reduction in dose intensity and a subsequent disparity in outcomes. This explanatory model, the Symptom Experience, Management, Outcomes and Adherence according to Race and Social determinants + Genomics Epigenomics and Metabolomics (SEMOARS + GEM), considers essential factors such as social determinants of health, clinician communication, symptoms and symptom management, genomics, epigenomics, and pharmacologic metabolism as contributory factors.

## Introduction

Breast cancer (BC) incidence is similar among black and white women [1], except for younger black women aged 45 and under, who have higher incidence rates [2]. Yet black women die from BC at a rate 42% higher than white women [1, 3] and are more frequently diagnosed at later disease stages and with aggressive triple-negative (estrogen, progesterone, HER2/neu) tumors [2]. This increase is particularly true in BC, confirmed when a meta-analysis reported a 1.22 odds ratio for a negative effect of African-American ethnicity on BC mortality [4]. These negative outcome differences persist after controlling for disease stage and tumor type, comorbidities, age, and insurance status, which leaves the underlying cause of this disparity unexplained [5, 6]. Receiving ≤ 85% of prescribed BC chemotherapy is associated with poor outcomes [7–9]. Racial disparity in cancer treatment is documented [10] and is a potential source of the racial variance in survival rates [3, 11–17].

Suboptimal adherence to chemotherapy treatment is a multifactorial problem, which involves much more than the patient herself. The International Society for Pharmacoeconomics and Outcomes Research defines medication compliance/adherence as “the degree or extent of conformity (most appropriately a percentage) to the recommendations about day-to-day treatment by the provider with respect to the timing, dosage, and frequency” [18]. Most often, studies investigating cancer treatment adherence focused on oral cancer treatments and have not included factors other than the patient’s role in adherence. The term “adherence” or “compliance” carries some traditionally pejorative connotations, implying that the choice to receive less than full-dose treatment is always patient initiated. In BC treatment, the choices regarding less than full adherence to prescribed BC intravenous chemotherapy are most often initiated by the clinical staff rather than the patient. Treatment decisions such as capping chemotherapy dosing at a body surface area (BSA) of 2.0 [19, 20] instead of treating to full body weight or the clinician’s subjective treatment decisions based on the categorization of certain women as “poor chemotherapy candidates” allowed a differential treatment approach that was potentially racially biased [21]. The standardization of chemotherapy dosing according to BSA without any or minimal cap for overweight and obese patients [22] and the standard use of national treatment guidelines in medical oncology [23] may now more closely regulate the clinician’s discretion during initial treatment prescription, limiting the clinician’s autonomy in prescribing nonstandard therapy or first cycle reduction. Perhaps these changes are reflected in recent studies, including our own, reporting on racial disparity in the initiation of chemotherapy. Slight to no racial disparity was found in the clinician’s prescription or the patient’s initiation of prescribed chemotherapy [24], but racial disparity in receiving full-dose, timely treatment across the chemotherapy continuum was noted [25–31].

The Symptom Experience, Management and Outcomes According to Race and Social determinants (SEMOARS) model was developed to address factors associated with the disparate receipt of chemotherapy. In this model, the exploration of these adjuvant BC chemotherapy receipt variables stresses the importance of the person within a social and environmental context. 

## The SEMOARS + GEM Model

**Fig. 1:**
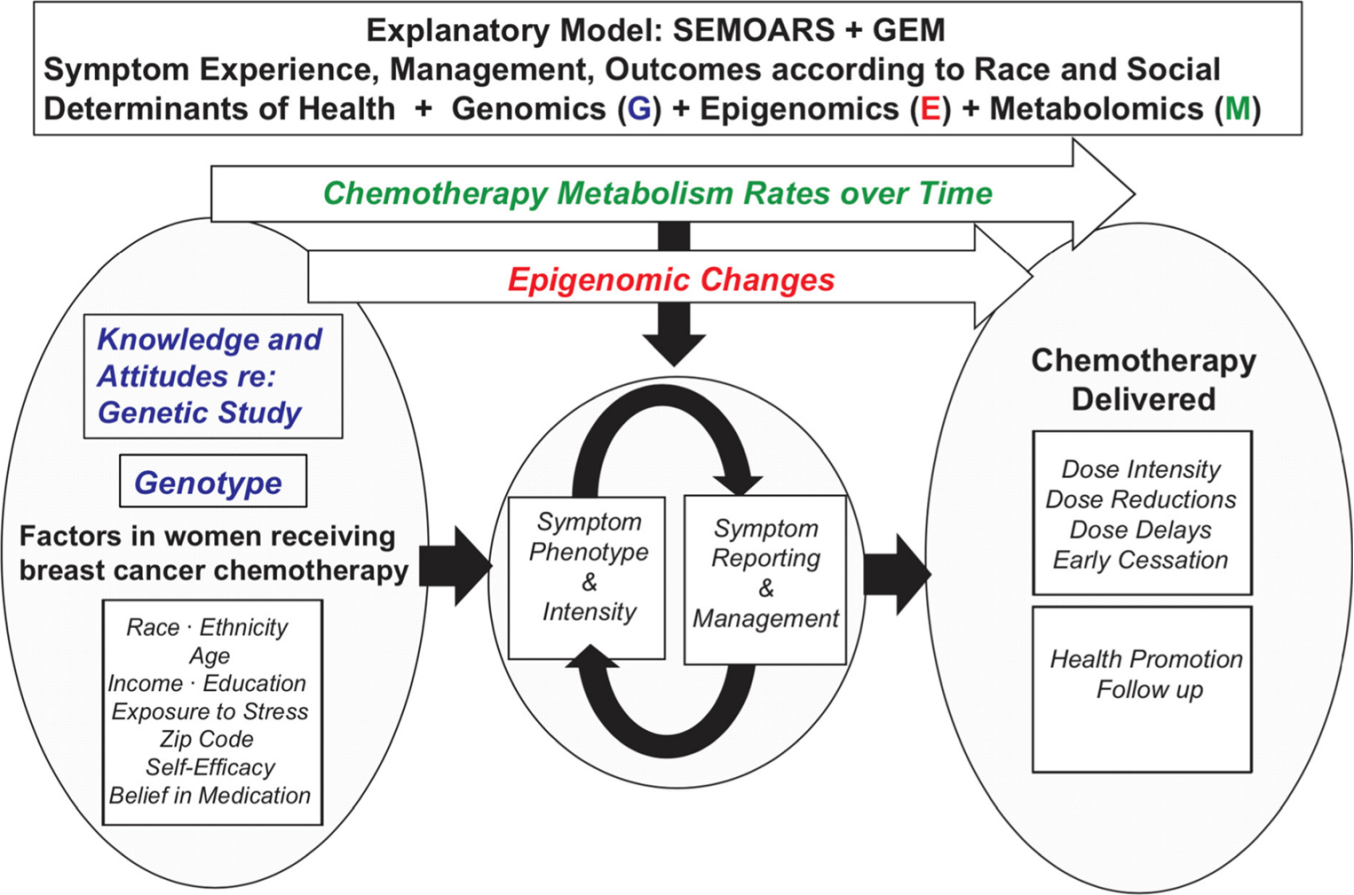
The SEMOARS + GEM explanatory model

The development of the SEMOARS model, with the addition of Genomics, Epigenomics, and Metabolomics (GEM) (Fig. 1), enables rasearchers to examine the variables contributing to the hypothesized explanatory model [32–41]. The SEMOARS + GEM model identifies crucial factors that contribute to racial disparity in dose reduction and early chemotherapy termination. These factors include symptom phenotype and intensity, symptom reporting and management, and social determinants of health. In addition, the biologic variables of genomics, epigenomics during BC chemotherapy, and chemotherapy metabolism are modeled. The purpose of this paper is to provide a presentation and explanation of this model with relevant science. We will explore each variable in the model (Fig. 1) and provide current supporting evidence (Table 1).

**Table 1:** Influence of social determinants of health, symptom experience, genomics and epigenomics on outcomes during breast cancer chemotherapy

**Age**	
Griggs et al. [26] Sample *N*=1403 Black 361 Low-acculturated Hispanic 186 High-acculturated Hispanic 183 Non-Hispanic white 673	Multivariable logistic regression o Increased age had lesser odds of receiving chemotherapy: OR 0.91 (95% CI 0.90–0.92)
Inwald et al. [42] Sample *N*=3463 Bavaria, Germany, no race data reported	Frequency o Women >70 years old were treated less frequently with chemotherapy + endocrine therapy (6.9%) than 50-69 years old women (28.3%)
Owusu et al. [43] Sample *N*=689 White 643 Minorities 46	Chi-square o Women >75 years old (9%) received less chemotherapy compared with 65 to ≤75 years (28%; *p* < .0001)
Sandy & Della-Fiorentina [8] Sample *N*=308 Sydney, Australia, no race data reported	Multivariable regression with backwards selection o Women age ≥ 65 years old had greater odds of having a dose reduction adjusted OR 8.36; 95% CI 2.40–29.08; *p*= .001
**Income/insurance**	
Griggs et al. [26] Sample *N*=1403 Black 361 Low-acculturated Hispanic 186 High-acculturated Hispanic 183 Non-Hispanic white 673	Multivariable logistic regression • Medicaid versus other insurance lesser odds of receiving chemotherapy OR 0.59; 95% CI, 0.37–0.95
Wells et al. [24] Sample *N*=99 Black 51 White 48	Logistic regression • Medicaid/no insurance versus private/private+Medicare* related to adherence to chemotherapy: *β*= –2.111; • Adjusted OR 0.121; *p*= .016
**Financial toxicity**	
Wheeler et al. [44]	Multivariable logistic regression predicted risk for black women compared with white
Sample *N*=2494	• Financial barrier adjusted risk difference 13.09 (SE 1.50) *p* < .001 • Insurance loss adjusted risk difference 3.37 (SE 0.83) *p* < .001
Black 49% White 51%	
**Education and symptoms**	
Prigozin et al. [45] Sample *N*=51	Pearson’s *r*: o Education and total symptom scores were inversely related *r*_s_= –0.41; *p* < .01
**Race and adherence**	
Griggs et al. [26] Sample *N*=1403 Black 361 Low-acculturated Hispanic 186 High-acculturated Hispanic 183 Non-Hispanic white 673	Multivariable logistic regression receipt of chemotherapy compared with non-Hispanic white women • Black women (ns) OR 0.83 95% CI 0.64–1.08 • Hispanic low acculturated women OR 2.00; 95% CI 1.31–3.04 • Hispanic high acculturated women OR 1.43; 95% CI 1.03–1.98
Wells et al. [24] Sample *N*=99 Black 51 White 48	Chi-square • No difference in adherence to chemotherapy between black and white patients: χ^2^= 2.627, *p*= .10
K. Smith et al. [46] Sample *N*=121 Black 21 White 98	Relative Risk • Modification of chemotherapy treatment in black versus white women: RR= 1.56; *p*= .04 • Black women received reduced cumulative doses of adjuvant chemotherapy: RR= 2.49; *p*= .03
Fedewa et al. [47] Sample *N*=107,587 White 69.75% Black 11.52% Hispanic 4.57% Asian 2.84% Other minorities 11.32%	Multivariate regression results • Greater risk of delay in black women (6.78% versus white 3.59%): 60-day delay RR= 1.36; 95% CI, 1.30–1.41 90-day delay RR= 1.56; 95% CI, 1.44–1.69 • Greater risk of delay in Hispanic women (6.91% versus white 3.59%): 60-day delay RR= 1.31; 95% CI, 1.23–1.39 90-day delay RR= 1.41; 95% CI, 1.26–1.59
Check et al. [48]	Generalized Linear Model Step-wise Regression with cancer-specific physical well-being and 1) race 2) clinical and demographics 3) interpersonal processes of care for black women:
Sample *N*=4002 (*N*=2740 for 6-month timepoint) Black 316 White 2672 Hispanic 498 Asian 516	• At baseline, interpersonal processes of care domains for compassion (*β=* 0.40; *p*= .02), elicited concerns (*β=* 0.59; *p*= .0009), and explained results (*β=* 0.46; *p*= .002) were positively associated with physical well-being and discrimination due to race was negatively associated (*β=* –0.58; *p*= .005) • Black and white women differences in physical well-being widened at 6 months (*β=* –0.99; *p*= .02)
Newman et al. [4]	Pooled meta-analysis of breast cancer mortality in black compared with white women:
Sample 14 studies *N*=52,474 Black 10,001 White 42,473	• Random effects for mortality OR 1.215; 95% CI 1.13–1.30 • Adjusted for socioeconomic status OR 1.27; 95% CI 1.17–1.38
**Symptom/severity**	
Simon et al. [49] Sample *N*=126 Black 27.8% White 65.1%	Independent sample t test chemotherapy induced peripheral neuropathy (CIPN) black women experienced and reported more CIPN compared with white women: • Sensory scale: 28.6 versus 14.4, *p* < .002 • Motor scale: 25.0 versus 15.6, *p* < .012 • Autonomic scale: 24.3 versus 13.4, *p* < .014 • Reported CIPN: 82.9% versus 67.1%
Yee et al. [32] Sample *N*=121 Black 100%	Pearson Correlation • Full dose chemotherapy at midpoint with: o Symptom distress at baseline r= 0.243; *p*= .007; mid-chemo course r= 0.187, *p*= .042; and completion r= 0.180, *p*= .050 o Total number of symptoms at baseline r= –0.225, *p*= .014 • Full dose chemotherapy at endpoint with: o Total number of symptoms at baseline r= 0.189; *p*= .039
Bandos et al. [50] Sample *N*=1512	Multivariable ordinal logistic regression • Women ≥50 were more likely to experience long term peripheral neuropathy OR 1.34; 95% CI 1.10–1.65; *p*=.005
Gnerlich et al. [51] Sample *N*=243,012	Cox regression • Younger (<40 years old) were more likely to die with Stage 1 (adjusted HR 1.44; 95% CI 1.27-1.64) or Stage 2 (adjusted HR 1.09; 95% CI 1.03–1.15) than women older than 40
Gaston-Johansson et al. [52] Sample *N*=30 Black 100%	Chi-square • Symptoms increased at midpoint of chemotherapy and then decreased or remained the same at completion. For example, worst pain χ^2^= 7.81, *p*= .027
Schneider et al. [53] Sample *N*=1779 African descent 213 European descent 1566	Logistic regression with Cox hazard ratio • Compared with other races, patients of African descent had increased risk of taxane-induced peripheral neuropathy (TIPN) grade 2-4 HR 2.1; *p*= 5.6 × 10^-16^ and grade 3-4 HR 2.6; *p*= 1.1 × 10^-11^
**Symptoms and race/ethnicity**	
Eversley et al. [34] Sample *N*=116 White 30% Black 30% Latina 25% Other minorities 15% Breast cancer survivors	Comparing race/ethnicity: o Latina reported more symptoms (*μ*= 2.5) than black (*μ*= 1.5) or white (*μ*= 1.2; *p* < .01) o Black (91%) and Latina (93%) reported more pain (white 54%; *p* < .001) o Latina (89%) reported more depressive symptoms compared with black (38%) and white (40%; *p* < .001) Least Squares Regression for total number of symptoms: o Income *β*= –0.397 *p*= .003 o Mastectomy *β*= 0.340 *p*= .005 o Chemotherapy *β*= 0.340 *p*= .026 o Latina *β*= 0.340 *p*= .004
Miaskowski et al. [54] Sample *N*=582 Breast, gastrointestinal, gynecological, or lung cancer patients undergoing chemotherapy	Latent Class Analysis yielded 3 trajectories for symptoms: o “All High” 13.9% of patients o Younger age F= 6.07; *p*= .002 (low versus moderate and high) o Less education F= 5.00; *p*= .007 (low versus moderate and high) o Minorities χ^2^= 8.81; *p*= .012 (low versus moderate and high) o Lower income KW= 22.81; *p* < . 0001 (low and moderate versus high) o Breast cancer χ^2^= 11.17; *p*= .083 o More comorbidities F= 38.99; *p* < .0001 (low versus moderate versus high) o Lower reported functional status F= 38.73; *p* < .0001 (low versus moderate versus high) o “Moderate” 50% of patients o “Low” 36.1% of patients o Fewer females χ^2^= 24.39; *p* < .0001 (low versus moderate and high) o More married/partnered χ^2^= 10.80; *p*= .005 (low versus high)
**Comorbidities and cancer**	
Leach et al. [55] Sample *N*=1527 Black 18.1% White 50.5% Other minorities 31.4%	Prevalence and Linear Regression: o Compared with breast cancer survivors, fewer comorbidities were reported by prostate cancer survivors *β=* –1.22; *p*= .0001; as well as colorectal cancer survivors *β=* –0.62; *p*= .0243 and ovarian cancer survivors *β=*–0.55; *p*= .042 o Compared with white cancer survivors, black cancer survivors reported fewer comorbidities *β=* –0.89; *p*= .0112 o Breast cancer survivors reported having experienced more comorbidities (5.8; 95% CI 5.4–6.2) than survivors of other cancers
**Comorbidity and adherence**	
Fedewa et al. [47] Sample *N*=107,587 Black 11.52% Hispanic 4.57% Asian 2.84% White 69.75% Other minorities 11.32%	Multivariate regression results Greater risk of delay compared with no comorbidity: • 60-day delay 1 comorbidity RR= 1.09; 95% CI, 1.04–1.14 ≥2 comorbidities RR= 1.32; 95% CI, 1.21–1.45 • 90-day delay 1 comorbidity RR= 1.13; 95% CI, 1.34–1.23 ≥2 comorbidities RR= 1.32; 95% CI, 1.10–1.60
**Comorbidity and survival**	
Braithwaite et al. [56] Sample *N*=1254 Black 416 White 838	Logistic regression with Cox hazard ratios • Hypertension increased risk of mortality after adjusting for age and race HR 1.33 95% CI 1.07–1.67
Klepin et al. [57] Sample *N*=329 Black 11% White 87% Other minorities 1% Unknown 1%	Multivariable logistic regression for overall survival • Total number of comorbidities HR 1.18; 95% CI 1.06–1.33; *p* < .01
**Beliefs and adherence**	
Gatti et al. [58] Sample *N*=275 Black 86.2% White 5.1% Other minorities 8.7%	Multivariable logistic regression on medication adherence in general • Negative beliefs about medication is a predictor of low adherence adjusted OR 2.12; 95% CI 1.3–3.7; *p*=.006
**Spirituality and patient-reported outcomes**	
Gaston-Johansson et al. [52] Sample *N*=30 Black 100%	Correlation • Negative religious coping correlated with psychological distress r= 0.6; *p* < .05, anxiety r= 0.51; *p* < .05, and depression r= 0.65; *p* < .01
**Interpersonal communication and mistrust**	
Sutton et al. [59] Sample *N*=210 Black 100%	Multiple linear regression • Low rating of chemotherapy communication was associated with greater medical mistrust high school or less *p*=.02
Tucker et al. [60] Sample *N*=298 Black 100%	Mediation analysis • Trust mediated the role of cultural sensitivity in the domains of provider competence/confidence, provider sensitivity/interpersonal skill, and provider respect/communication with patient satisfaction
Jiang et al. [33] Sample *N*=101 Black 100%	Multiple linear regression • Perceived better physician interpersonal communication was positively associated with beliefs in the necessity of chemotherapy *β=* 0.057; *p*= .007
**Genomics and taxane-induced peripheral neuropathy (TIPN)**	
Schneider et al. [61] Sample *N*=213 Black 100%	Gene-based case control statistical analysis (SKAT) • *SET binding factor 2* (*SBF2*) was associated with TIPN *p*= 4.35 × 10^−6^
Hertz et al. [62] Sample *N* = 411 White discovery cohort 209 Black replication cohort 107	Log-rank test and Cox proportional hazards • In European-American discovery cohort, CYP2C8*3 genotype increased risk of grade 2+ neuropathy for each allele HR =1.95; 95% CI 1.06–3.58; *p* = .031 • In African-American replication cohort, no homozygotes were found, but one allele of CYP2C8*3 increase TIPN risk HR = 3.30; 95% CI 1.04–10.45; *p* = .043
Baldwin et al. [63] Sample *N* = 1126 White discovery cohort 855 Black replication cohort 154 White replication cohort 117	Ordinal logistic regression o In the white (European) discovery cohort, *FGD4* was associated with TIPN HR 1.57; 95% CI 1.30–1.91; *p* = 2.6 × 10^−6^ o The white replication cohort was similar HR 1.72; 95% CI 1.06–2.80; *p* = .013 o The black replication cohort was also associated HR 1.93; 95% CI 1.13–3.28; *p* = 6.7 × 10^−3^
Abraham et al. [64] Sample *N* = 1303 samples from several trials White 100%	Unconditional logistic regression and likelihood ratio test o *ATP-binding cassette, subfamily B (ABCB1)* was associated with decreased odds of TIPN OR 0.47; 95% CI 0.28–0.79; *p* = .004 o *Tubulin Beta 2A Class IIa (TUBB2A)* was also associated with increased odds of TIPN OR 1.80; 95% CI 1.20–2.72; *p* = .005
Apellaniz-Ruiz et al. [65] Sample *N* = 146 White 100%	Cumulative dose analysis and additive model o *Ephrin Receptor A5 (EPHA5)* was associated with TIPN HR 2.3; 95% CI 1.6–3.9; *p* = .0074 o *Ephrin Receptor A6 (EPHA6)* was associated with TIPN HR 1.9; 95% CI 1.2–2.9; *p* = .0063 o *Ephrin Receptor A8 (EPHA8)* was associated with TIPN HR 1.9; 95% CI 1.1–3.2; *p* = .0012
Boso et al. [66] Sample *N* = 113 White 100%	Multivariate logistic regression o *Excision repair cross complementation group 1 (ERCC1)* was associated with TIPN *p* = .006
**Epigenomics and chemotherapy**	
Smith et al. [67.] Sample *N* = 61 Black 25 White 36	Linear regression (MethLAB) o CpG sites with change in methylation after chemotherapy versus no chemotherapy included o cg26077811 *β* = −.074, *p* = 3.65 × 10^−9^ o cg18942579 *β* = −.161, *p* = 1.65 × 10^−8^ o cg12054453 *β* = −.154, *p* = 2.75 × 10^−8^ o cg16936953 *β* = −.168, *p* = 3.26 × 10^−8^ o cg05438378 *β* = −.089, *p =* 7.78 × 10^−8^ o cg25446789 *β* = −.085, *p* = 7.84 × 10^−8^ o cg01409343 *β* = −.138, *p* = 9.88 × 10^−8^ o cg13518625 *β* = −.051, *p* = 9.98 × 10^−8^

Where studies categorized race as other or nonwhite (likely grouped due to the sample size), we used the terms minorities or other minorities

*ns*, not significant; *OR*, odds ratio; *HR*, hazard ratio; *CI*, confidence interval; *RR*, relative risk; *SE*, standard error; *SD*, standard deviation; *β*, beta; *μ*, mean; *KW*, Kruskal-Wallis; *χ*^2^ , chi-square; *RR*, risk ratio; “white” was used in some cases when European ancestry was indicated

*Health insurance variable was a surrogate for income/socioeconomic class

**Only measured number of sessions, not dose. Adherence divided into 100% attendance or less than 100% and was defined by patient factors: missed appointments, cancellations, no shows, etc.; no delays or discontinuations by the provider were noted

### Social Determinants of Health

The model begins with the patient (within the context of her social and physical environment) initiating BC chemotherapy. Social determinants of health associated with increased symptom experience and intensity are considered integral and specific to each patient. Race/ethnicity, age, income, education, zip code, allostatic load, comorbidity, and self-efficacy/belief in prescribed medication are social determinants of health that may be associated with increased symptoms resulting in dose reductions, chemotherapy holds, and early therapy cessation [68–74]. The following sections provide a review of evidence of these associations to date.

### Race/Ethnicity

Racial and ethnic differences in treatment delivery and symptoms are documented [75–77]. Black women experience more chemotherapy delays compared with white women [47, 78]. Symptoms may be a causative factor. For example, minority women describe more symptom intensity and distress BC treatment [79, 80]. In a recently completed study of 140 black women receiving adjuvant BC chemotherapy, nearly all (99%) black women initiated chemotherapy, and almost 40% received a reduction in dose intensity, early cessation, or delay associated with symptoms and cancer-related distress [32].

### Age

Evidence regarding the influence of age on symptom distress is contradictory. Older women are more likely to receive a lower chemotherapy dose intensity, due to fewer prescriptions and more dose reductions than younger women, with a subsequent decrease in overall survival [8, 42, 43]. Most studies adjust for age in their analyses, and when examined as a factor related to symptoms, age produced mixed results. Older age was associated with increased long-term peripheral neuropathy in docetaxel regimens [50] and more overall toxicity, such as chemotherapy-induced bone marrow toxicities [81]. Conversely, younger women experience an increase in symptoms related to cognitive function [82]. Miaskowski et al. examined factors across multiple tumor types associated with increased symptom distress during cancer chemotherapy and found younger age, female sex, low social support, and socioeconomic status to be characteristic of the symptom grouping for greater symptom severity, suggesting multiple factors, including age, are related to symptoms experienced [54].

### Income/Education/Zip Code

Indicators of socioeconomic status, including income, education level, and zip code, affect women’s experience of BC chemotherapy and overall treatment. Lower-income women are more likely to report symptoms after treatment [34] as well as a financial burden, and black women report a greater financial burden than white women after controlling for socioeconomic status [44]. Education level, which is often correlated with socioeconomic status, was inversely related to chemotherapy symptoms, with better-educated women reporting a lower symptom burden [45] and being less likely to receive a chemotherapy dose reduction [22]. Additionally, black women with less education were more likely to report perceived discrimination and disparities in their care [59].

Zip code may be used as a surrogate measure of socioeconomic status, such as income, education, and employment, in addition to the geographical region. For example, Griggs et al. found that when compared with the Northeastern region of the USA, patients in the Southern region had greater odds of receiving a reduction in their chemotherapy dose [22]. Financial, educational, and geographic factors influence symptoms and BC treatment intensity.

### Allostatic Load

Allostatic load is an algorithmic risk factor representing cumulative stress exposure causing persistent, severe psychological and physical symptoms for any illness, specifically cancer. Geronimus et al. used the term “weathering” to characterize the effect of cumulative stress from multiple stressors on US blacks in their residential, occupational, and other environments [83]. Thus, among black and low-income women, there is increasing concern about the impact of a lifetime of accumulated stress on illness outcomes, including BC outcomes [84, 85]. The impact of the full range of childhood and cumulative adult-life stress exposure has not yet been studied in relation to cancer-related symptoms.

### Comorbidity

Black women with BC have more comorbid conditions [86] than white women has implications for BC outcomes. For example, hypertension accounted for 30% of racial survival disparity for one BC cohort [56]. An 18% increased risk of death was observed with each additional comorbid condition [57]. Comorbidities may interfere with treatment and are associated with chemotherapy delays [47, 87]. A meta-analysis concluded that patients with comorbidities had lesser odds of receiving chemotherapy and greater odds of toxicity [88]. The precise means by which comorbidities increase symptom incidence and distress and influence chemotherapy intensity is not clear.

### Beliefs and Communication

The belief that medication is necessary and efficacious, in addition to the concern over possible harmful effects, can influence whether a patient will carry out a prescribed treatment [58]. Concern may result from mistrust among black patients in a traditionally white health care system or belief among black women that health care providers are not sufficiently culturally sensitive to address specific concerns [59, 60, 89, 90]. Communication is essential to establishing trust in the provider-patient relationship and was negatively correlated with medical mistrust among black women with BC [59]. The communication patterns between clinician and patient, described as the patient centeredness of care (PCC), coded and scored through a 23-item checklist, may be an important explanation for racial differences in communication during BC clinical visits. Rosenzweig’s team described a prospective, comparative pilot study qualitatively coded for PCC during the clinical visit of women undergoing BC chemotherapy and compared by race. Twenty-four clinical visits were recorded in a sample of five black and five white women undergoing BC chemotherapy. Overall for each PCC item, the mean clinician visit scores for black women were higher (worse PCC) than the mean clinician visit scores for white women. Significant differences were found in 27% of the PCC items. The higher scores were evident for three of the four subscales “Invest in the Beginning,” “Elicit the Patient’s Perspective,” and “Demonstrate Empathy” [91].

## Symptom Phenotype and Intensity

For all women, once the chemotherapy dosing is calculated and initiated, follow-up doses may be decreased, held, or discontinued if patients exhibit symptoms of toxicity. There is a pattern during chemotherapy that symptoms increase from pre-chemotherapy to mid-therapy but stabilize after chemotherapy treatment midpoint to completion [32, 52], suggesting a symptom tolerance among patients. Associations between the ability to receive ≥ 85% of the prescribed treatment course and symptom distress, severity, and the total number of symptoms at pre-chemotherapy are reported [32]. Minority patients were more likely to belong to the high-symptom group when symptom severity was categorized into low, moderate, and high [54]. Other variables to consider in racial symptom and treatment disparity include baseline genomics and temporal epigenomic changes that may be associated with symptom phenotype and treatment response.

## Genomics and Epigenomics of Symptoms and Chemotherapy Metabolomics

Though social determinants of health are factors related to disparity in dosing and completion of chemotherapy, they do not fully account for the disparity in BC symptoms and ability to receive the full dose of chemotherapy in black women compared with white women. Genomic variation may help to explain a portion of these differences. For example, taxane-based chemotherapy used in BC treatment has a highly variable drug response and symptom profile and is metabolized through the cytochrome P450 system. Variations in cytochrome P450 genes *CYP3A4*, *CYP3A5*, and *CYP2C8*, as well as transporter genes *ABCB1*, *ABCB2*, and *SCLO1B3*, would likely result in individual differences in drug metabolism [92]. Genotype variations may result in an increase or decrease in patients’ symptoms, based upon the drug and the gene’s role in metabolism. CYP2C8*3 was associated with grade 2+ neuropathy in European- and African-Americans treated with paclitaxel, but African-Americans with the variant had greater odds of developing taxane-induced peripheral neuropathy, and no homozygotes for the variant were observed [62].

There is considerable variability in absorption, distribution, metabolism, and excretion (ADME) of drugs. These differences can be explained by genetic variation in ADME-related genes [93]. ADME-related genetic variability often differs across populations [94] and helps to explain the link between ancestry and variable chemotherapy drug response [95]. Li et al. observed greater diversity in ADME genes for the African-American population compared with European and African populations [96], predisposing African-Americans to more variable drug response. Gene variations may alter drug metabolism by activating or inactivating a medication, activating or inactivating a drug’s metabolite, affecting the medication’s transport, or affecting the drug’s intended target [97]. Concomitant medications may also facilitate or interfere with drug metabolism.

Genes not directly involved in drug metabolism may affect symptoms experienced among diverse ancestries. For example, taxane-induced peripheral neuropathy (TIPN) is a common symptom with known genomic associations. Variants in the Charcot-Marie-Tooth (CMT) disease gene, *SBF2*, were predictors of TIPN in black patients [61]. Another CMT gene, *FGD4*, was associated with paclitaxel-induced peripheral neuropathy in patients of European or African ancestry [63]. *ABCB1* (noted as a transporter above), *ERCC1*, *TUBB2A*, and *EPHA5/6/8* were associated with neuropathy in European samples [64–66]. Most genome-wide databases revealed focus exclusively on European populations, rather than African, Asian, and Latin American, which underscores the gap in genome-wide research and importance of recruitment for all ancestral populations in research [98, 99].

**Fig. 2:**
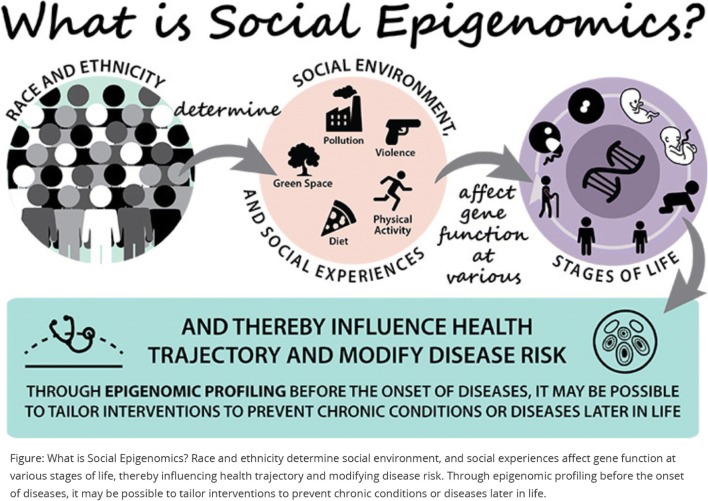
The role of social determinants and epigenomics in health and disease*.* Figure used with permission from NIH (https://epi.grants.cancer.gov/epigen.html)

Genomic variation in ancestry and drug metabolism genes are not the only factors associated with the development of toxicities. Epigenetic changes, perhaps those caused by chemotherapy, other cancer treatments, or social determinants of health, may also increase symptoms (Fig. 2). DNA methylation, the addition of a methyl group to the nucleotide cytosine, is one type of epigenetic change which potentially affects gene expression by changing the gene’s activity (increase or decrease). For example, an increase in inflammatory markers was found in patients with BC following a decrease in DNA methylation post-chemotherapy, indicating that DNA methylation mediated a relationship between chemotherapy and inflammatory biomarkers [67]. The study of epigenetic change resulting from chemotherapy and its influence on drug response and symptoms is an emerging field.

In summary, the SEMOARS + GEM explanatory model for disparity among black women with BC examines psychosocial, clinical, and biological factors impacting treatment delivery, symptoms, and outcomes. Testing each aspect of this model and determining the unique contribution of each component to overall BC treatment disparity is critically important to understanding the most relevant actionable targets for ensuring treatment equity.

## Discussion

We presented evidence for the development of the SEMOARS + GEM model of disparity of BC treatment in black women. This comprehensive model, supported by previous research, describes a process that transpires during a BC diagnosis and treatment. Black women bring their life experiences and characteristics, in the form of social determinants of health and genomic profiles, at the time of diagnosis. These factors influence the patient’s symptom experience including symptom phenotype, intensity, reporting to clinicians, and subsequent clinician management. Resultant dose alterations or early cessation may occur. Epigenetic changes and chemotherapy metabolism over time may moderate the symptom experience.

Many of the variables discussed in this paper, for example, education and socioeconomic status and similarly BMI and comorbidities, are typically correlated with one another. Instead of examining race and age as predictors, many studies control for them statistically. Additionally, most study samples do not include a large enough black population to draw inferences, and few focus solely on black women.

A disparity in outcomes for black women with BC has been well-established, but no one factor explains the issue in its entirety. Outcomes for black women are influenced by social determinants of health [100]. Barsevick et al. reported that social determinants such as education, unemployment, marital status, age, comorbidity, and medical mistrust were factors in post-treatment burden for survivors [101]. All of these factors are examined in the SEMOARS + GEM model. Potentially pertinent factors, not examined in depth, are those addressed in the US government’s public health initiative, Healthy People 2020. According to Healthy People 2020, social determinants of health encompass economic stability, education, social and community context, health and health care, and neighborhood and built environment [102]. The SEMOARS + GEM model somewhat superficially measures many of these concepts, though perhaps not as in-depth as prescribed by Healthy People 2020.

Symptom phenotype and intensity experienced by black women are more pronounced than in white women, resulting in an inability of more black women to receive the entire chemotherapy treatment and leading to poorer outcomes. Additionally, Zannas et al. provided evidence of the impact of social determinants of health on epigenetic aging in an African-American cohort, confirming that in addition to genomics, stressors leading to physiologic changes should be further studied [103].

The SEMOARS + GEM model will add in-depth descriptive work to find actionable targets, which will inform further implementation and translation into clinical practice guidelines. Should identification of genomic associations ensue, a precision health care strategy may be routinely implemented into clinical care to identify at-risk patients for early symptom management in order to tailor chemotherapy treatments to the patient. Providing an individualized protocol for all patients with BC, including factors specific to black women, will offer improved symptom management. Biological ancestral differences in chemotherapy metabolism and epigenomics suggest that variations influence symptom and treatment outcome differences.

The SEMOARS + GEM model includes known factors that influence a black woman’s disparate BC treatment experience to inform future interventions to improve the ability to receive complete, effective BC chemotherapy.
